# African American Race and Prevalence of Atrial Fibrillation:A Meta-Analysis

**DOI:** 10.1155/2012/275624

**Published:** 2012-04-05

**Authors:** Marlow B. Hernandez, Craig R. Asher, Adrian V. Hernandez, Gian M. Novaro

**Affiliations:** ^1^Department of Internal Medicine, Cleveland Clinic Florida, Weston, FL 33331, USA; ^2^Department of Cardiology, Cleveland Clinic Florida, 2950 Cleveland Clinic Blvd, Weston, FL 33331, USA; ^3^Department of Quantitative Health Sciences, Cleveland Clinic, Cleveland, OH 44195, USA; ^4^Quantitative Research Division, BioEstadistica, S.C., Monterrey, NL 66260, Mexico

## Abstract

*Background*. It has been observed that African American race is associated with a lower prevalence of atrial fibrillation (AF) compared to Caucasian race. To better quantify the association between African American race and AF, we performed a meta-analysis of published studies among different patient populations which reported the presence of AF by race. *Methods*. A literature search was conducted using electronic databases between January 1999 and January 2011. The search was limited to published studies in English conducted in the United States, which clearly defined the presence of AF in African American and Caucasian subjects. A meta-analysis was performed with prevalence of AF as the primary endpoint. *Results*. In total, 10 studies involving 1,031,351 subjects were included. According to a random effects analysis, African American race was associated with a protective effect with regard to AF as compared to Caucasian race (odds ratio 0.51, 95% CI 0.44 to 0.59, *P* < 0.001). In subgroup analyses, African American race was significantly associated with a lower prevalence of AF in the general population, those hospitalized or greater than 60 years old, postcoronary artery bypass surgery patients, and subjects with heart failure. *Conclusions*. In a broad sweep of subjects in the general population and hospitalized patients, the prevalence of AF in African Americans is consistently lower than in Caucasians.

## 1. Introduction

Atrial fibrillation (AF) is the most common type of persistent arrhythmia in the United States, with prevalence likely to rise and a burgeoning national healthcare cost burden [1]. Despite its familiarity, the well-known associated risk factors such as advancing age, hypertension [2], heart failure [3], diabetes [4], and larger body mass index [5] fail to fully account for the burden of risk leading to AF [6]. Recently, race and related genetic variants have been suggested as novel susceptibility factors contributing to the development of AF [7]. Along these lines, it has been observed that African American race confers a lower risk of developing AF compared to white race despite having a greater risk burden for AF [8, 9]. Although these epidemiologic findings seem consistent, a counterpoint contends that an underdiagnosis of AF among different racial groups may be the contributing factor to account for the AF disparity seen in African Americans [10]. Investigators have questioned the impact of race on the epidemiology of AF based on issues relating to the imperfect methodology for AF detection, coupled with the suspicion that AF is unequally diagnosed among racial groups [10, 11].

 The aim of the present study was to systematically quantify the association between African American race and prevalence of AF in a wide range of patient populations. To address this objective, we performed a meta-analysis of available published studies among different patient subgroups which addressed the presence of AF in subjects of African American and Caucasian race.

## 2. Methods

### 2.1. Search Strategy

A detailed literature search was conducted using electronic databases, including PubMed, MD Consult, and ISI Web of Science for the period between 1999 and January 2011. All designs of studies (observational, cross-sectional, case-control, and cohort studies) on the epidemiology of AF were considered. The following search terms were used:* atrial fibrillation, blacks, African American, African continental ancestry group*,* race, ethnicity, and prevalence*. Combinations of these keywords or search terms were used to expand the sample size for the analysis; the search strategy for PubMed is shown in the appendix. The search was limited to studies published in the English language and conducted in the United States. For all initially retrieved studies, study abstracts were reviewed and screened, followed by full-text article review and appraisal for eligibility. A manual search of reference citations from the selected studies was also considered for inclusion. If multiple articles were published for a single study, the latest publication was selected. Studies were appraised and selected by 2 reviewers (G.M.N., M.B.H.). Disagreements on inclusion/exclusion were solved by consensus or by consultation with a third reviewer (C.R.A.).

### 2.2. Study Criteria

Included studies must have defined AF as AF or atrial flutter found in more than one outpatient diagnosis of AF, more than 1 electrocardiogram with AF, more than 1 hospitalization for AF, or more than 1 electrocardiogram with AF in hospital after cardiac surgery. The studies must have clearly defined African Americans or non-Hispanic blacks and Caucasians or non-Hispanic whites (as members of one racial group in the United States, resp.), and reported their AF and non-AF cases in each group. This allowed for data from the various studies to be merged. The studies must also have included consecutive (nonselected) samples, with strong external validity relative to the specific study question. Exclusion criteria for the studies included study population <150 patients; single gender studies; studies which compared blacks to other groups or whites to other groups, but not whites to blacks specifically; studies which focused on patients with stroke; studies which did not focus on epidemiological associations but rather were aimed at answering a specific clinical question. The majority of these studies were eliminated because of sampling techniques, which though necessary for clinical research, introduce sampling bias into epidemiological analysis.

### 2.3. Data Extraction

The following data were extracted from the original studies: (1) characteristics of study and study subjects (age range inclusion, type of study population, method of AF ascertainment, and states where subjects were enrolled), (2) sample sizes, (3) number of African Americans with AF, (4) number of African Americans without AF, (5) number of Caucasians with AF, and (6) number of Caucasians without AF. Study differences as to collection or report of data were noted when appropriate. Two investigators (G.M.N., M.B.H.) reviewed and extracted the data from the included studies.

### 2.4. Study Quality Assessment

We systematically assessed key points of study quality proposed by the MOOSE collaboration [12]. These key points were (1) clear identification of study population; (2) clear definition of outcome and outcome assessment; (3) independent assessment of outcome parameters (i.e., ascertainment of outcomes done by researchers other than the ones involved in the study); (4) no selective loss during followup; (5) important confounders and/or prognostic factors identified. Each point was rated as Yes/No. If the description was unclear, we considered this as “No”.

### 2.5. Statistical Analysis

Our study followed the Preferred Reporting Items for Systematic Reviews and Meta-analyses (PRISMA) statement [13]. The association between race and AF was shown as odds ratios (OR) and their 95% confidence intervals (CI). ORs were reported as adjusted for confounders when available. When incidence rates were reported, prevalence rates were calculated in order to be consistent with other included studies. Prevalence was estimated by dividing the number of total AF in each race by the corresponding number of total individuals within that cohort. A DerSimonian and Laird random effects model [14] was used due to the expected heterogeneity across studies; the inverse variance method was used to calculate pooled ORs. Statistical heterogeneity was evaluated with the Cochran Chi-square (*χ*2) and quantified with the *I* ^2^ statistic (low is <25%, moderate 25–50%, high >50%) [15]. Subgroup analyses were prespecified to deal with heterogeneity and were considered secondary analyses. Subgroups were defined as general public age >50 years; hospitalized or elderly; postcoronary artery bypass grafting surgery; heart failure. Publication bias was evaluated by testing for funnel plot asymmetry and the Egger's regression test. Significance was set at a *P* value of less than 0.05. All statistical calculations were made using Medcalc 2011 software (Mariakerke, Belgium), the RevMan 5.1 software (Cochrane Collaboration, London, UK), and the *metafor* package of R (http://www.metafor-project.org/).

## 3. Results

**Figure 1:**
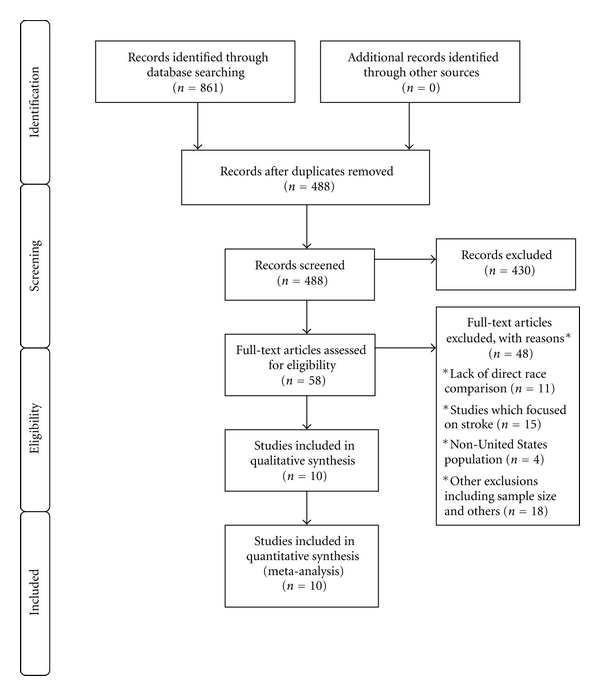
PRISMA flow diagram for selection of studies.

After an initial search identified 488 studies, application of inclusion and exclusion criteria selected 10 studies [7, 16–24] with a total of 1,031,351 subjects determined to be suitable for the meta-analysis (Figure 1). Most of the excluded studies were based on absence to address AF prevalence. Further, many of the studies which addressed AF prevalence did not report race-specific data. The number of subjects by race involved in the studies ranged from 50 to 519,714. Of the 10 identified studies, 4 studies with 812,200 subjects were conducted in the general population [7, 16, 17, 22], 2 studies with 1,536 subjects in patients with heart failure [18, 19], 1 with 211,915 subjects in patients on hemodialysis [20], 1 with 2,123 subjects in hospitalized patients [21], and 2 with 3,577 subjects in patients postcoronary artery bypass grafting surgery [23, 24]. The characteristics of the 10 studies are shown in Table 1.

The study by Go et al. [16] had a sample which included persons other than Caucasians and African Americans which were eliminated from the meta-analysis. Further, as there was limited data on race for those <50 years old and given the very low prevalence of AF in younger patients, the analyzed patients were those >50 years old (A. S. Go, personal communication, April 26, 2011). It should also be noted that the Go et al. [16] study failed to show differences in AF prevalence among patients 50–59 years of age. In the study by Alonso et al. [17], incidence rates were reported. Based on the study data, prevalence rates were calculated in order to be consistent with other included studies. In addition to reporting data on hemodialysis patients, the report by Winkelmayer et al. [20] noted that white race compared to black race was found to be an independent predictor of AF in the nondialysis chronic kidney disease population. In the study by Marcus et al. [7], we used only the data from the Cardiovascular Health Study (CHS) cohort, as they also included the Atherosclerosis Risk in Communities study [17] as part of their meta-analysis. Most of the studies had an overall good quality (Table 2).

### 3.1. Meta-Analysis

**Figure 2:**
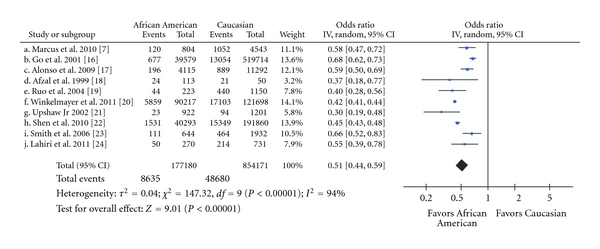
Meta-analysis of the association between African American race and prevalence of atrial fibrillation. The squares represent the odds ratio and their sizes are proportional to the study sample; the horizontal lines represent 95% confidence intervals (CI).

**Figure 3:**
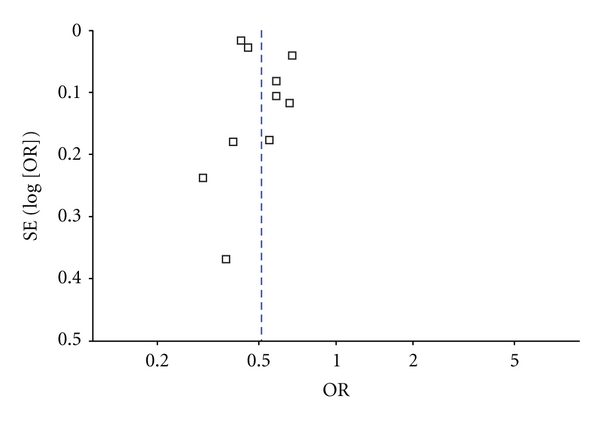
Funnel plot.

All 10 studies reported a substantially lower prevalence of AF among African Americans, as compared to Caucasians (Figure 2, Table 3). African American race was significantly associated with a lower prevalence of AF, as compared to Caucasian race (OR 0.51, 95% CI 0.44 to 0.59, *P* < 0.001). Heterogeneity of the effect measures was high (Cochran's *Q* = 147.3, *P* < 0.001, *I* ^2^ = 94%). The association was similar when using the Risk Ratio (RR) as a measure of association (RR 0.56, 95% CI 0.49 to 0.63). The subgroup analyses are summarized in Table 4. We noted that African American race was significantly associated with a lower prevalence of AF in the general public, those hospitalized or elderly, patients postcoronary artery bypass surgery, and subjects with heart failure. There was no evidence of publication bias (Figure 3; Egger's test *P* = 0.3).

## 4. Discussion

In this meta-analysis involving over a million individuals, we have observed consistently that African Americans manifest a significantly lower prevalence of AF compared to Caucasians. In fact, African American race appears protective of AF with about a 50% decrease in risk compared to Caucasian race. Our analysis goes on to demonstrate that the lower risk of AF among African Americans was seen among various patient subgroups such as the general public, hospitalized patients, those with heart failure, and those postcoronary artery bypass surgery.

 With regard to the racial risk associated with AF, it is suggested that the biologic underpinnings for the observed disparity in AF prevalence are genetically mediated. Using ancestry informative markers, Marcus et al. observed that increasing European ancestry, as opposed to African ancestry, among African Americans was associated with an increased risk of AF [7]. This finding was at odds with the greater AF risk factor burden found among African Americans, suggesting a rather dominant effect of the racial-genetic influence. Further insight into the race-mediated effects of AF risk comes from data on left atrial size. Echocardiographic cohort studies have noted larger left atrial sizes in Caucasians as compared to African Americans even after adjusting for significant confounders [25]. In a study of hypertensive males, whites had a greater left atrial size than African Americans, even after adjustments for covariates such as age, body mass index, and left ventricular mass [26]. The authors purported that conceivably, among blacks, an increase in left atrial wall thickness may limit enlargement of the left atrium, a hypothesis which requires testing.

 A contrary perspective put forth to explain the lower prevalence of AF among African Americans relative to Caucasians is the existence of an ascertainment bias [10]. This hypothesis suggests that the ability to detect AF may be different across racial groups [10, 11]. It may be that by applying more sensitive methods for AF detection, the racial disparity would be attenuated [11]. It is also plausible to consider that African Americans do not present similarly with AF symptoms or do not access medical care equally often. However, the inclusion of studies in this meta-analysis that assessed serial electrocardiograms and hospitalized patients on continuous electrocardiographic monitoring such as those with post-operative AF after coronary bypass surgery make the likelihood of ascertainment bias much less likely. Furthermore, it has been suggested that the higher stroke rate seen in African Americans is incompatible with an apparent lower prevalence of AF. This viewpoint is not supported by the finding of many studies that demonstrate that African Americans with stroke, compared to whites, are more often hypertensive and have a very low etiological fraction of AF [27].

 Our study has several limitations which are worthy of mention. Since we did not have access to patient level data, our analysis relied on published unadjusted aggregate results. While some studies did not specifically report the number of positive and negative cases, we had to calculate the number based on reported incidence or prevalence rates and general study population demographics. Despite heterogeneity between the included studies, the odds ratios reported were fairly consistent between studies. Indeed, we feel that the use of random effects model in our analysis adequately accounts for the AF prevalence rate difference between Caucasians and African Americans across a diverse population. In this regard, the limited availability to expand the subgroup populations, despite being a prespecified analysis, represents a study shortcoming. Finally, it should be recognized that although the meta-analysis was limited to studies conducted in the United States, it remains possible that a portion of African American subjects may be of European or Caribbean descent, rendering the group more genetically diverse. Similarly we limited the Caucasian groups to whites of non-Hispanic ethnicity in an effort to restrict the comparison to a more homogeneous racial-ethnic group.

## 5. Conclusions

In our meta-analysis of a broad sweep of subjects in the general population and hospitalized patients with various cardiac and noncardiac conditions, the prevalence of AF in African Americans is consistently lower than Caucasians. Although explanations for this finding are only speculative, they do suggest that despite a higher risk burden in African Americans, a genetically mediated protective effect may be contributory.

## Figures and Tables

**Table 1:** Characteristics of included studies in the meta-analysis.

First Author Year (Ref)	Study Years	AA, *n *	Caucasian, *n *	Age, yrs	Population	Study design	Enrollment states
Marcus et al. 2010 [7]	1989-1999	804	4,543	>65	Older population	Cohort	15 U.S. states**, Washington D.C.
Go et al. 2001 [16]	1996-1997	39,579	519,714	>50*	General population	Cross-sectional	California
Alonso et al. 2009 [17]	1987–2004	4,115	11,292	>55*	General population	Cohort	Maryland,
							Minnesota,
							Mississippi,
							N. Carolina
Afzal et al. 1999 [18]	1996	113	50	>35*	Heart failure	Cohort	Michigan
Ruo et al. 2004 [19]	1999-2000	223	1,150	>35*	Heart failure	Cohort	California
Winkelmayer et al. 2011 [20]	1992–2006	90,217	121,698	>30*	Hemodialysis	Cohort	United States
Upshaw Jr. 2002 [21]	1996–1998	922	1,201	>20	Hospitalized patients	Cohort	Georgia
Shen et al. 2010 [22]	2008	40,293	191,860	>60	Older population	Cross-sectional	California
Smith et al. 2006 [23]	1993–2005	644	1,932	>25*	Post-CABG	Case control	Ohio
Lahiri et al. 2011 [24]	2004–2008	270	731	>25*	Post-CABG	Cohort	Michigan

*More than 99% of patients were older than the stated age, according to reported study demographics. It should be noted that in patients less than 50 years of age, the prevalence of AF was similar across racial lines in the general population.

**States included Alabama, California, Connecticut, Florida, Georgia, Illinois, Iowa, Maryland, Minnesota, North Carolina, Oregon, Pennsylvania, Tennessee, Texas, and Washington.

AA = African American; CABG = coronary artery bypass grafting.

**Table 2:** Study quality summary.

First author (Ref)	Clear identification of study population	Clear definition of outcome and outcome assessment	Independent Assessment of outcome parameters	No selective loss during followup	Important confounders and/or prognostic factors indentified	Adjusted odds ratio (specific adjusters)
Marcus et al. [7]	Yes	Yes	Yes	Yes	Yes	0.75 (age, gender)
Go et al. [16]	Yes	Yes	Yes	Yes	Yes	Not provided
Alonso et al. [17]	Yes	Yes	Yes	Yes	Yes	0.59 (age, gender)
Afzal et al. [18]	Yes	Yes	No	Yes	Yes	Not provided
Ruo et al. [19]	Yes	Yes	Yes	Yes	Yes	0.51 (age, gender, Comorbid conditions, medications)
Winkelmayer et al. [20]	Yes	Yes	Yes	Yes	Yes	Not provided
Upshaw Jr. [21]	Yes	Yes	Yes	Yes	Yes	Not provided
Shen et al. [22]	Yes	Yes	Yes	Yes	Yes	0.49 (age, gender)
Smith et al. [23]	Yes	Yes	Yes	Yes	Yes	0.64 (unclear)
Lahiri et al. [24]	Yes	Yes	Yes	Yes	Yes	0.54 (age, gender, comorbid conditions)

**Table 3:** Prevalence of atrial fibrillation among African American and Caucasians in included studies.

First Author (Ref. #)	Year	AA with AF	(%)	Caucasian with AF	(%)
Marcus et al. [7]	2010	120/804	15%	1052/4,543	23%
Go et al. [16]	2001	677/39,579	1.7%	13,054/519,714	2.5%
Alonso et al. [17]	2009	196/4,115	4.8%	889/11,292	7.9%
Afzal et al. [18]	1999	24/113	21%	21/50	42%
Ruo et al. [19]	2004	44/223	20%	440/1,150	38%
Winkelmayer et al. [20]	2011	5,859/90,217	6.5%	17,103/121,698	14%
Upshaw Jr [21]	2002	23/922	2.5%	94/1,201	7.8%
Shen et al. [22]	2010	1,531/40,293	3.8%	15,349/191,860	8.0%
Smith et al. [23]	2006	111/644	17%	464/1,932	24%
Lahiri et al. [24]	2011	50/270	19%	214/731	29%

AA = African American; AF = atrial fibrillation.

**Table 4:** Subpopulation analysis showing the effect of African American race on prevalence of atrial fibrillation.

Subpopulatio*n* (Ref. #)	AA AF%	Caucasian AF%	Sample size	OR (95% CI)	*P* value
Age > 50 years [16, 17]	2.0%	2.6%	574,700	0.66 (0.61–0.71)	<0.001
Hospitalized or elderly [7, 21, 22]*	3.9%	8.4%	239,623	0.46 (0.36–0.59)	<0.001
Post-CABG surgery [23, 24]	18%	25%	3,577	0.62 (0.52–0.76)	<0.001
Heart failure [18, 19]	20%	38%	1,536	0.39 (0.29–0.54)	<0.001

*Utilized the Mantel-Haenszel method, carrying out a random effects model, given that the test for heterogeneity is statistically significant; *Q* = 8.7242; DF = 2; *I* ^2^ = 77.1%; *P* = 0.0128. AA = African American; AF = atrial fibrillation; CABG = coronary artery bypass grafting; CI = confidence interval; OR = odds ratio.

## Appendix

### PubMed Search Strategy

(“atrial fibrillation" [MeSH Terms] OR (“atrial” [All Fields] AND “fibrillation” [All Fields]) OR “atrial fibrillation” [All Fields]) AND ((“african continental ancestry group” [MeSH Terms] OR (“African” [All Fields] AND “continental” [All Fields] AND “ancestry” [All Fields] AND “group” [All Fields]) OR “african continental ancestry group” [All Fields] OR “blacks” [All Fields]) OR (“african americans” [MeSH Terms] OR (“african” [All Fields] AND “americans” [All Fields]) OR “african americans” [All Fields] OR (“african” [All Fields] AND “american” [All Fields]) OR “african american” [All Fields]) OR (“continental population groups” [MeSH Terms] OR (“continental” [All Fields] AND “population” [All Fields] AND “groups” [All Fields]) OR “continental population groups” [All Fields] OR “race” [All Fields]) OR (“ethnology” [Subheading] OR “ethnology” [All Fields] OR “ethnicity” [All Fields] OR “ethnology” [MeSH Terms] OR “ethnicity” [All Fields] OR “ethnic groups” [MeSH Terms] OR (“ethnic” [All Fields] AND “groups” [All Fields]) OR “ethnic groups” [All Fields])) AND (“epidemiology” [Subheading] OR “epidemiology” [All Fields] OR “prevalence” [All Fields] OR “prevalence” [MeSH Terms]).
